# Developmental dynamics of crop morphology, microbiota, and mucosal immunity in chickens

**DOI:** 10.1186/s40104-026-01495-8

**Published:** 2026-09-08

**Authors:** Fei Zhang, Mengya Lu, Hongbo Jiang, Xiangyu Wang, Yanli Liu, Xiaojun Yang, Zhouzheng Ren

**Affiliations:** College of Animal Science and Technology, Northwest A&F University, Yangling, Shaanxi 712100 China

**Keywords:** Crop, Development, Histomorphology, Lueyang Black-boned chicken, Microbiota

## Abstract

**Background:**

The crop is a unique anterior digestive organ in poultry that functions in temporary feed storage and lactic acid bacteria–dominated fermentation, thereby interacting with the gastrointestinal tract to influence gut health and feed efficiency. However, its developmental characteristics and functional establishment across the entire growth cycle remain poorly understood.

**Results:**

A slow-growing chicken breed (Lueyang Black-boned chicken) was used to characterize dynamic changes in crop histomorphology, mucosal immune status, and microbiota from hatch to adulthood (d 1, 14, 28, 42, 56, 70, 98, and 140; *n* = 10 per time point). Crop development was divided into two stages. From d 1 to d 42, the crop underwent structural and microbial establishment. This stage was characterized by a slow increase in crop weight, progressive thickening and stabilization of the muscular and mucosal layers, and a gradual increase in secretory immunoglobulin A (sIgA) levels. Genes involved in mucin synthesis and modification were markedly upregulated during early development (d 1 to d 14). Meanwhile, the microbiota shifted from environmentally associated facultative anaerobes to lactic acid bacteria–dominated communities (*Lactobacillus*, *Ligilactobacillus*, and *unclassified Bacilli*). Correspondingly, predicted microbial functions shifted from colonization-related pathways to carbohydrate, fatty acid, and butyrate metabolism. From d 42 to d 140, the crop entered a stage of structural expansion and microbial restructuring. Crop weight increased rapidly, and the mucosal layer exhibited fluctuations, while sIgA levels continued to increase and stabilized. During d 42 to d 56, fluctuations in mucosal thickness were accompanied by reduced mucin sialylation. At the same time, lactic acid bacteria populations shifted, with a decrease in *Ligilactobacillus* and an increase in *unclassified Bacilli*, followed by stabilization of the microbial community. Functional prediction indicated enrichment of membrane transport systems and diverse nutrient metabolism pathways.

**Conclusion:**

This study demonstrates that the early post-hatch period establishes the crop’s basic functions, whereas the growing period is characterized by crop expansion and further maturation of the microbial community. These findings provide a theoretical basis for targeted interventions in the crop to improve poultry production performance.

**Graphical Abstract:**

**Figure:**
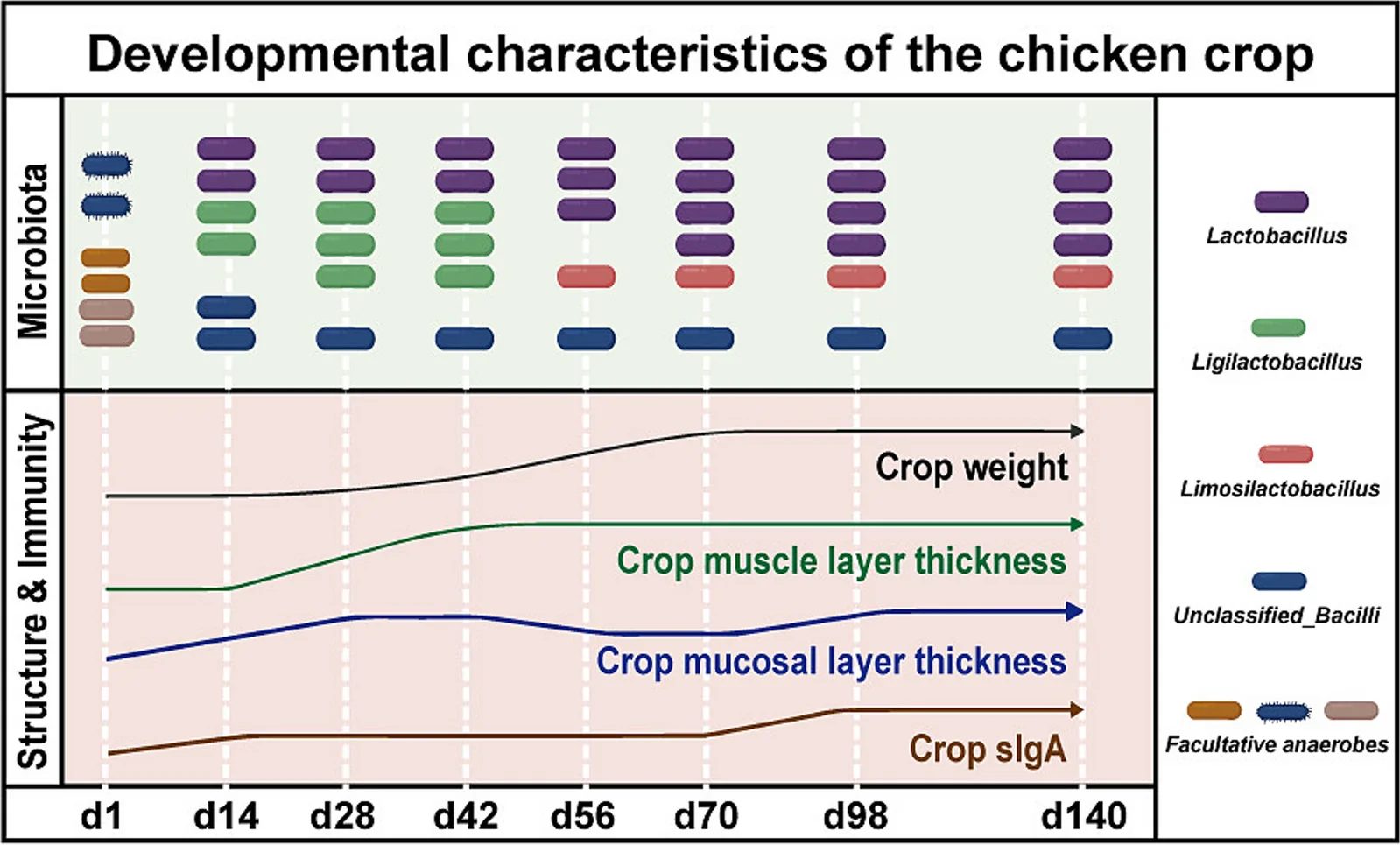

**Supplementary Information:**

The online version contains supplementary material available at https://doi.org/10.1186/s40104-026-01495-8.

## Introduction

The digestive system of poultry consists of functionally specialized organs that operate in a coordinated manner to influence production performance [1, 2]. The crop is a unique digestive organ in poultry that is involved in feed storage and microbial fermentation [3–6]. Structurally, the crop is highly elastic and resembles the esophagus. In practice, crop fullness is commonly used to assess feeding status. Previous studies by our team have confirmed that genetic selection for high body weight leads to an increase in crop weight and enhanced fermentation potential of the microbiota in chickens [7]. The storage function of the crop can also influence feed efficiency by regulating the flow rate of digesta along the gastrointestinal tract [8, 9]. The crop microbiota, dominated by *Lactobacillus* spp., ferments feed to produce metabolites such as lactic acid and short-chain fatty acids [5, 10], which help maintain microbial homeostasis and may exert beneficial effects on downstream gut health and nutrient absorption [11–13]. In addition, secretory immunoglobulin A (sIgA) produced by the crop mucosa plays an important role in preventing pathogen invasion and maintaining local immune homeostasis [6, 14]. Collectively, these findings highlight the important role of the crop in maintaining gastrointestinal health and improving nutrient utilization in poultry.

The functions of digestive organs exhibit dynamic developmental patterns during growth, and understanding these processes is essential for improving poultry production performance [15]. However, current studies on the crop have mainly focused on specific physiological conditions or single time points, with emphasis on microbial composition or digesta flow characteristics [16–19]. In contrast, the developmental trajectory of the crop throughout the production cycle, particularly the coordinated changes in histomorphology, microbiota, and mucosal immune, remains poorly understood. Therefore, this study used a slow-growing chicken breed with a long production cycle and well-defined developmental stages (Lueyang Black-boned chicken) as a model. By integrating histological, microbiological, and immunological analyses, we systematically characterized crop development across different growth stages. This study aims to identify critical windows for crop functional development and to provide a theoretical basis for targeted nutritional and microbial interventions to optimize gastrointestinal homeostasis and improve production performance.

## Materials and methods

### Chemical composition of the diet

The diets were formulated according to the recommended level of the Chinese Feeding Standard of Chicken (NY/T 33–2004) [20]. The dietary metabolizable energy (ME) was calculated as the sum of the product of ingredient inclusion levels and their corresponding ME values:

$$\mathrm{ME}_{\mathrm{diet}} = \Sigma (\mathrm{X}_{{i}} \times \mathrm{ME}_{{i}})$$

where X_*i*_ represents the inclusion level of ingredient *i* (kg/kg diet), and ME_*i*_ is the metabolizable energy value of ingredient *i* obtained from the 32^nd^ edition of the Tables of Feed Composition and Nutritive in China [21]. Premix was excluded due to negligible energy contribution. The crude protein (N × 6.25) content was determined using the Kjeldahl method 990.03 [22] with a nitrogen analyzer (CNS-2000, LECO Corporation, St. Joseph, MI, USA). With reference to the previous methodology, the actual phosphorus content in the diet was analyzed using a spectrophotometer (UV-2700, Shimadzu Corporation, Kyoto, Japan) in accordance with GB/T 6437–2018 [23], while the calcium content was measured using flame atomic absorption spectrophotometry (Zeenit700P, Analytik Jena AG, Jena, Germany) according to GB/T 6436–2018 [24]. The samples were ashed at 600 °C for 12 h using a muffle furnace, and the minerals were determined using inductively coupled plasma mass spectrometry (7400, Thermo Fisher Scientific, Waltham, MA, USA). The calculated nutritional values in Table 1 referred to the 32^nd^ edition of the Tables of Feed Composition and Nutritive in China [21].

**Table 1:** Ingredient composition of diet (as-fed basis), %

Ingredients	Content		Nutrient (analyzed, unless noted)	Content	
	**d 1–42**	**d 43–140**		**d 1–42**	**d 43–140**
Corn	62.00	60.00	Metabolizable energy (calculated, kcal/kg)	2.78	2.76
Soybean meal (43%)	19.00	12.00	Crude protein (calculated/analyzed)	19.87/19.75	16.31/16.12
Soybean protein concentrate	7.50	/	Calcium (calculated/analyzed)	1.08/1.20	0.97/0.92
Corn husk	/	15.00	Total phosphorus (calculated/analyzed)	0.65/0.65	0.57/0.54
Soy oil	0.5	0.60	Available phosphorus (calculated)	0.39	0.30
Wheat bran	5.69	6.51	Lysine (calculated)	1.33	0.79
Coarse limestone	0.8	1.80	Methionine (calculated)	0.46	0.38
Dicalcium phosphate	1.5	0.60	Threonine (calculated)	0.78	0.59
Salt	0.30	0.30			
Choline chloride (60%)	0.05	0.05			
Glutamic acid residue	/	2.00			
L-Lysine (70%)	0.45	0.31			
OH-Methionine (88%)	/	0.12			
DL-Methionine	0.04	/			
L-Threonine	0.06	/			
L-Isoleucine	0.06	0.02			
L-Tryptophan	/	0.04			
Premix^a^	2.00	/			
Premix^b^	/	0.60			
Solid mold inhibitor	0.05	0.05			
Total	100.00	100.00			

^a ^Premix provided per kg of diet: Vitamin A, 10,080 IU; Vitamin D_3_, 3,600 IU; Vitamin E, 63 mg; Vitamin K_3_, 3 mg; Vitamin B_1_, 6 mg; Vitamin B_2_, 11 mg; Vitamin B_6_, 14 mg; Vitamin B_12_, 0.05 mg; Biotin, 0.39 mg; Niacin, 92 mg; Folic acid, 2 mg; Pantothenic acid, 27 mg; Manganese, 104 mg; Zinc, 116 mg; Iron, 219 mg; Iodine, 0.54 mg; Copper, 19 mg; Selenium, 0.40 mg; Sulfur, 531 mg; Cobalt, 0.18 mg

^b ^Premix provided per kg of diet: Vitamin A, 10,080 IU; Vitamin D_3_, 3,600 IU; Vitamin E, 65 mg; Vitamin K_3_, 3 mg; Vitamin B_1_, 5 mg; Vitamin B_2_, 11 mg; Vitamin B_6_, 15 mg; Vitamin B_12_, 0.08 mg; Biotin, 0.38 mg; Niacin, 100 mg; Folic acid, 2 mg; Pantothenic acid, 28 mg; Manganese, 98 mg; Zinc, 94 mg; Iron, 238 mg; Iodine, 0.60 mg; Copper, 15 mg; Selenium, 0.40 mg; Sulfur, 612 mg; Cobalt, 0.08 mg

### Chickens and sampling

A total of 100 one-day-old male Lueyang Black-boned chickens were used in this study. All chickens were individually numbered at day 1 for subsequent random selection. All chickens were reared at the Institute of Poultry–Crop Integrating Systems, Northwest A&F University. Chickens were housed at five per cage (depth × width × height = 80 cm × 40 cm × 40 cm) from d 1 to d 42 and individually caged (45 cm × 35 cm × 45 cm) from d 43 to d 140. Chickens were fed a starter diet from d 1 to d 42 and a grower diet from d 43 to d 140, with ad libitum access to feed and water (Table 1). At each time point (d 1, 14, 28, 42, 56, 70, 98, and 140), 10 chickens were randomly selected using a computer-generated random number method. The sample size was determined based on previous poultry developmental studies to balance biological variability, statistical power, and animal welfare. The selected chickens were clinically healthy and had body weights consistent with the age-specific reference values established from long-term records of this breed maintained by our research group. They were then weighed and humanely euthanized, and serum and crop samples were collected. Environmental conditions, including temperature, ventilation, and lighting, followed the standard recommendations of the Shaanxi Engineering Research Center of Lueyang Black Chicken. Rearing temperature was gradually reduced from 38–36 °C (d 1 to d 3) to 23–20 °C at 6 weeks of age (decreasing by 2–3 °C per week), and then maintained. Relative humidity was controlled at 60%–70% (d 1 to d 14), 50%–60% (d 15 to d 21), and 50%–55% thereafter. The lighting program started with 24 h light (d 1 to d 4), then gradually decreased to 12 h light:12 h darkness, which was maintained. The crop tissue was weighed, and the following samples were collected: (1) crop tissue (stored at −80 °C for qPCR and sIgA analyses); (2) crop tissue (fixed in 4% paraformaldehyde for histological analysis); and (3) crop irrigation solutions (stored at −80 °C for 16S rRNA analysis). Serum samples were used for biochemical analysis.

### Serum biochemical assay

Blood sample of each chicken were collected in separate glass tubes (2 mL per chicken, via the left wing vein). Serum samples were obtained from the blood samples and were stored at −80 °C for future analysis. Serum levels of total protein, glucose, triglyceride, total cholesterol, uric acid, albumin, alanine aminotransferase, aspartate aminotransferase, and total bilirubin were analyzed at Yangling Demonstration District Hospital (Yangling, Shaanxi, China) using a fully automatic biochemical analyzer (7180, Hitachi High-Technologies, Tokyo, Japan).

### Crop histomorphology

For microscopic examination, crop tissue samples (approximately 1 cm × 1 cm) were collected from the center of the pouch of each chicken, fixed in 4% paraformaldehyde (Catalog No. BL539A, Biosharp Life Sciences, Hefei, China) for 48 h, and processed for paraffin embedding. Cross-sections (5 µm) were prepared and stained with hematoxylin and eosin (H&E) (Catalog No. G1076, Servicebio, Wuhan, China) and alcian blue–periodic acid Schiff (AB-PAS) (Catalog No. G1285, Servicebio, Wuhan, China) for histomorphometric evaluation. Imaging and measurements were performed using a positive fluorescence microscope (Eclipse E100, Nikon, Tokyo, Japan). For histomorphological analysis, the thickness of the muscle layer (H&E) and mucosal layer (AB-PAS) was measured at six positions per section, and mean values were calculated [25].

### Crop tissue mRNA expressions

Crop tissue samples were stored at −80 °C for mRNA expression analysis. RT-PCR was performed as previously described [26]. Briefly, total RNA was extracted using Trizol reagent (Catalog No. AG21102, Accurate Biology, Changsha, China), and RNA concentration and purity were assessed with a NanoDrop spectrophotometer (2000c, Thermo Fisher Scientific, Wilmington, DE, USA). Qualified RNA was reverse-transcribed into cDNA using the PrimerScript RT Reagent Kit (Catalog No. AG11706, Accurate Biology, Changsha, China). qPCR was conducted with the SYBR Premix Ex Taq kit (Catalog No. AG11701, Accurate Biology, Changsha, China) on a QuantStudio 5 system (Thermo Fisher Scientific, Waltham, MA, USA) under the following conditions: 95 °C for 30 s, followed by 40 cycles of 95 °C for 5 s and 60 °C for 30 s. Primer sequences are listed in Table 2 and include genes related to cell proliferation, apoptosis, and mucin modification. Relative mRNA expression levels were normalized to β-actin using the 2^−ΔΔCt^ method.

**Table 2:** Sequences of primers for quantitative real-time PCR assay

Genes	Sequences (5'→3')	Accession number	Fragments sizes, bp
*ACTB*	F: ATTGTCCACCGCAAATGCTTC	L08165	113
	R: AAATAAAGCCATGCCAATCTCGTC		
*WNT3*	F: GAAGCTGCGAGGTCAAGACT	NM_001081696.1	265
	R: TTGCACGTTCTGTCCCTTGT		
*CASP3*	F: CACAGGCAAAGGCTGTCAAA	NM_204,725.1	63
	R: TCCGTGCAATTTTCCTTGCT		
*MUC2*	F: CCCTGGAAGTAGAGGTGACTG	XM_001234581.3	143
	R: TGACAAGCCATTGAAGGACA		
*C1GALT1*	F: TCCGACCTTGCGGTTTCATT	XM_040681142.1	88
	R: ACCCATACGGACGGAGATGA		
*GCNT1*	F: GCAACGCTAGTGGTGGTGAT	XM_004949175.5	144
	R: CCAAGTTCCAAGCCCCAGTG		
*GCNT2*	F: GAAGCGGTCAGGGGACTCT	XM_425064.7	124
	R: ACCGGCCCCATTCTGTCTAA		
*GCNT3*	F: AATGCCCTCCGGCATTTCTG	XM_046905796.1	99
	R: CACAGAGCAAGCTGGACCAC		
*CHST2*	F: TCTGCTTCCTGCTTATGGGGA	XM_046899692.1	105
	R: GGCAAGCAAACCATGCACTC		
*ST6GALNAC1*	F: GATCCTGACAGTGTGCCGAG	XM_040649461.2	82
	R: TGGCTTCCGAGGTCCTCTTC		
*ST6GAL1*	F: GTTGGGGTACATCCACGCAG	XM_046923641.1	137
	R: AAAGCATCTCCCGCGATTCC		
*ST3GAL4*	F: CCGCGTGGAAGAACTGTGAG	XM_046903793.1	147
	R: CGAGTACCACACCATGACCA		
*FUT8*	F: TGGTTCCTGGCGTTGGATTA	NM_001004766.2	100
	R: ATGATCTGGGTGCTCACTGTC		
*FUT3*	F: TATGAGCACTTCTTGCCCCC	XM_025144347.3	112
	R: GCTGGTACTTCTCGGTGTCC		
*ST3GAL1*	F: TTCGCCTTCGTACTCATCACG	XM_046919030.1	77
	R: GGATCCCAAGTGGTCATGGTC		

*ACTB* β-Actin, *CASP3* caspase 3,* C1GALT1* Core 1 beta-1,3-galactosyltransferase, *CHST2* Carbohydrate sulfotransferase 2, *FUT3* Fucosyltransferase 3, *FUT8* Fucosyltransferase 8, *GCNT1* Glucosaminyl (N-acetyl) transferase 1, *GCNT2* Glucosaminyl (N-acetyl) transferase 2, *GCNT2* Glucosaminyl (N-acetyl) transferase 3, *MUC2* Mucin 2, *ST3GAL1* ST3 beta-galactoside alpha-2,3-sialyltransferase 1, *ST3GAL4* ST3 beta-galactoside alpha-2,3-sialyltransferase 4, *ST6GAL1* ST6 beta-galactoside alpha-2,6-sialyltransferase 1, *ST6GALNAC1* ST6 N-acetylgalactosaminide alpha-2,6-sialyltransferase 1, *WNT3* Wnt family member 3

### Crop tissue sIgA

The concentration of sIgA in crop tissues was analyzed using Chicken-specific double antibody sandwich sIgA ELISA kit (Catalog No. RX600526CH, Quanzhou Ruixin Biotechnology Co., Ltd., Quanzhou, China) [27]. Approximately 0.1 g of crop tissue was weighed into 2-mL centrifuge tubes. Then, 900 μL of pre-cooled phosphate buffered saline (0.01 mol/L, pH = 7.4) and two ceramic beads were added. Samples were homogenized for eight cycles of 30 s at 65 Hz at 4 °C. Afterward, the samples were centrifuged at 4 °C for 15 min at 5,000 × *g*, and the supernatant was collected. ELISA was conducted according to the manufacturer's instructions. After the ELISA reaction, absorbance was measured at 450 nm using a microplate reader (Synergy HT, BioTek Instruments, Winooski, VT, USA). Then, the concentration of sIgA was calculated according to the standard curve. The standard curve was established by plotting the concentrations of the standards (2,400, 1,200, 600, 300, 150, 75, and 0 ng/mL) against their corresponding optical density (OD) values. A simple linear regression model was established using computer software. The concentrations of the target analyte in the samples were then calculated from the corresponding OD values according to the standard curve.

Standard curve:

$$y = 1401.5x - 81.993; {R}^2 = 0.9957$$

*y*: sIgA concentration (ng/mL); *x*: OD 450 nm value.

### 16S rRNA analysis of crop digesta microbiota

Crop irrigation solutions were collected from each chicken, and total microbial genomic DNA was extracted using the E.Z.N.A.^®^ Soil DNA Kit (Catalog No. T10-100, MJYH, Shanghai, China). The V3–V4 region of the bacterial 16S rRNA gene was amplified with primer pairs 338 F (5′-ACTCCTACGGGAGGCAGCAG-3′) and 806R (5′-GGACTACHVGGGTWTCTAAT-3′) using a T100 Thermal Cycler (Bio-Rad Laboratories, Hercules, CA, USA). Raw sequencing reads were deposited in the NCBI Sequence Read Archive under BioProject accession number PRJNA1336469.

After demultiplexing, sequences were quality-filtered with fastp (v0.19.6) [28], merged using FLASH (v1.2.11) [29], and denoised with the DADA2 plugin [30] in QIIME2 (v2020.2) [31], yielding amplicon sequence variants (ASVs) at single-nucleotide resolution. To normalize sequencing depth, samples were rarefied to 20,000 reads, retaining an average Good’s coverage of 97.9%. Taxonomic assignment was performed with the QIIME2 naïve Bayes classifier against the SILVA 16S rRNA database (v138).

Bioinformatic analyses were conducted on the Majorbio Cloud platform (https://cloud.majorbio.com). Rarefaction curves and alpha diversity indices (observed ASVs, Chao1 richness, Shannon index, and Good’s coverage) were calculated using Mothur v1.30.1 [32]. Community similarity among samples was assessed by principal coordinate analysis (PCoA) based on Bray–Curtis dissimilarity using the Vegan v2.5-3 package, with significance tested by analysis of similarities (ANOSIM). Differentially enriched taxa were identified using linear discriminant analysis effect size (LEfSe) [33] with an LDA score > 2 and *P* < 0.05. The crop microbiota typing analysis was performed based on genus-level relative abundance profiles. Jensen–Shannon distance was calculated, followed by partitioning around medoids (PAM) clustering. The optimal number of clusters (K) was determined using the Calinski–Harabasz index. When K ≥ 3, between-class analysis was performed for visualization of clustering results [34].

Functional prediction of microbial communities was performed using PICRUSt2 [35]. ASV representative sequences were placed into a reference tree using EPA-NG and GAPPA, normalized by gene copy numbers with Castor, and annotated using MinPath to predict gene family profiles mapped to Kyoto Encyclopedia of Genes and Genomes (KEGG) pathways and Gene Ontology (GO). Differentially enriched pathways were identified using the limma package, and pathways with an adjusted *P* < 0.05 were considered significantly enriched.

### Statistical analysis

Data were analyzed using SPSS version 25.0 (IBM Corp., Chicago, IL, USA), with the individual chicken considered as the experimental unit. Histomorphological parameters, serum biochemical indices, gene expression levels, and sIgA concentrations were first tested for normality using the Shapiro–Wilk test and for homogeneity of variances using Levene's test. Normally distributed data were analyzed using one-way ANOVA followed by Tukey's multiple comparison test, whereas non-normally distributed data were analyzed using the Kruskal–Wallis test. Comparisons of microbial alpha diversity indices and the relative abundances of microbial taxa were performed using the Kruskal–Wallis test. Beta diversity of the crop microbiota was evaluated using ANOSIM based on Bray–Curtis dissimilarity. Predicted microbial functional profiles were analyzed using the limma package to identify differentially enriched KEGG pathways and GO terms, with adjusted* P* < 0.05 considered statistically significant. Results are presented as the mean ± standard error of the mean (SEM). For analyses performed in SPSS, statistical significance was set at *P* < 0.05, whereas trends were considered at 0.05 < *P* ≤ 0.10.

## Results

### Body weight and serum biochemistry

**Fig. 1:**
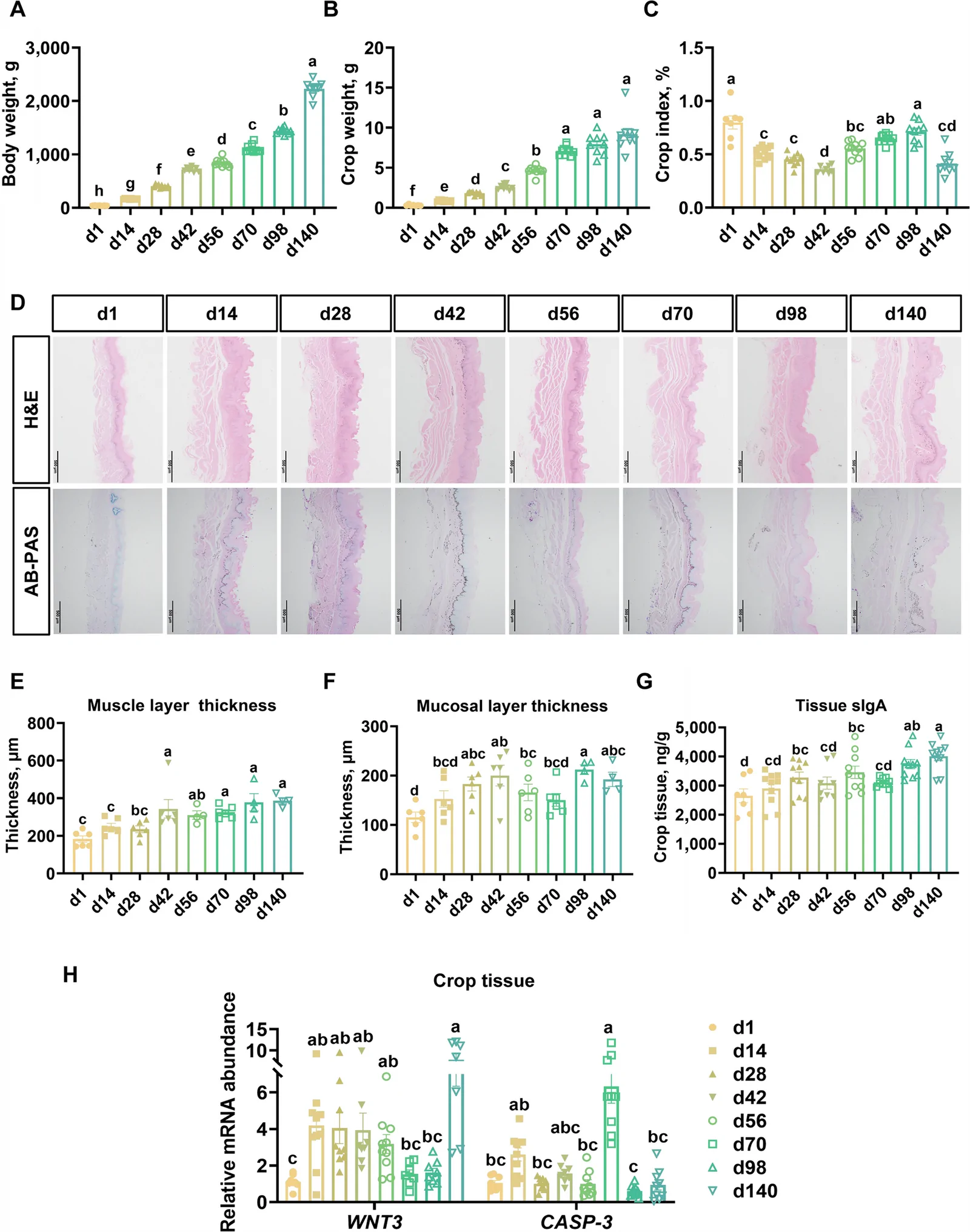
Development of crop histomorphology and mucosal immune status in the chicken crop across different ages. **A** Body weight of chickens. **B** Crop weight. **C** Crop index (relative to body weight). **D** Histological observations of crop tissue by H&E and AB-PAS staining. **E** Thickness of the crop muscle layer (μm). **F** Thickness of the crop mucosal layer (μm). **G** sIgA level in crop tissue. **H** Relative mRNA expression of proliferation genes (*WNT3*) and the apoptosis gene (*CASP3*). Data are expressed as mean ± SEM. Statistical significance was assessed by one-way ANOVA. Different letters (**a**–**h**) indicate significant differences among the treatment groups (*P* < 0.05). AB-PAS, Alcian blue–periodic acid Schiff; *CASP3*, Caspase 3; H&E, Hematoxylin and eosin; SEM, Standard error of the mean; sIgA, Secretory immunoglobulin A; *WNT3*, Wnt family member 3

Body weight increased continuously from d 1 to d 140, with a greater increase observed during d 42 to d 140 than during d 1 to d 42 (Fig. 1A; *P* < 0.05). Serum total cholesterol (TC) and triglyceride (TG) concentrations were highest at d 1, decreased significantly during the period from d 1 to d 14, and then remained relatively stable thereafter (*P* < 0.05; Table 3). Serum glucose concentration was significantly higher during d 14 to d 28 than at the other ages (*P* < 0.05; Table 3). Significant differences were also observed among ages for serum total protein (TP), uric acid (UA), albumin (ALB), alanine aminotransferase (ALT), aspartate aminotransferase (AST), and total bilirubin (TBIL), although these parameters did not exhibit a consistent age-related pattern of change (*P* < 0.05; Table 3).

**Table 3:** Serum biochemistry of the chickens

Item	Ages								SEM	*P*-value
	**d 1**	**d 14**	**d 28**	**d 42**	**d 56**	**d 70**	**d 98**	**d 140**		
TP, g/L	36.29^c^	30.25^d^	33.82^c^	33.93^c^	36.04^c^	36.36^c^	39.82^b^	44.80^a^	0.62	< 0.001
GLU, mmol/L	11.75^b^	13.47^a^	13.78^a^	11.04^bc^	11.68^b^	9.99^c^	10.76^bc^	11.99^b^	0.22	< 0.001
TC, mmol/L	3.25^a^	0.46^c^	0.62^c^	0.36^c^	1.22^b^	0.75^bc^	0.66^c^	0.38^c^	0.11	< 0.001
TG, mmol/L	13.64^a^	3.20^bc^	2.90^c^	2.91^c^	3.77^b^	2.74^c^	3.06^b^	3.18^b^	0.37	< 0.001
UA, mmol/L	294.00^abc^	318.10^ab^	308.10^abc^	363.13^a^	223.30^bc^	298.00^abc^	186.60^c^	219.20^bc^	14.74	0.035
ALB, g/L	13.57^d^	15.06^c^	13.56^d^	15.28^bc^	14.44^ cd^	15.65^abc^	16.56^ab^	16.77^a^	0.20	< 0.001
ALT, U/L	8.09^a^	6.94^ab^	6.01^bc^	6.83^b^	5.46^c^	5.54^c^	5.18^c^	5.86^bc^	0.17	< 0.001
AST, U/L	233.69^ab^	255.38^a^	211.57^bc^	233.90^ab^	228.69^ab^	194.37^c^	211.50^bc^	211.50^bc^	3.84	< 0.001
TBIL, μmol/L	204.51^a^	98.48^ cd^	97.67^d^	98.65^bcd^	100.43^b^	99.56^bc^	99.03^bcd^	100.09^bc^	3.57	< 0.001

*ALB* Albumin, *ALT* Alanine aminotransferase, *AST* Aspartate aminotransferase, *GLU* Glucose, *SEM* Standard error of the mean, *TBIL* Total bilirubin, *TC* Total cholesterol, *TG* Triglyceride, *TP* Total protein, *UA* Uric acid

^a–d ^Means within a row lacking a common superscript differ (*P* ≤ 0.05)

### Age-related changes in crop histomorphology

Crop histomorphology was evaluated based on the organ index and the thickness of the muscular and mucosal layers. Crop weight increased significantly with age and reached a plateau after d 70 (*P* < 0.05), with a greater gain observed during d 42 to d 70 than during d 1 to d 42 (Fig. 1B). Correspondingly, the crop index decreased significantly from d 1 to d 42 (*P* < 0.05), increased during d 42 to d 70 (*P* < 0.05), and declined again at the later growth stage (d 98 to d 140) (Fig. 1C). Further histological analysis of the crop is shown in Fig. 1D. The thickness of the muscular layer increased significantly from d 1 to d 42 (*P* < 0.05) and remained relatively stable thereafter. The thickness of the mucosal layer increased from d 1 to d 28 (*P* < 0.05), fluctuated between d 42 and d 70, and became stable from d 98 onward. Gene expression analysis showed that the expression level of the proliferation-related gene *WNT3* was significantly upregulated during the period of crop weight gain (*P* < 0.05), whereas the expression level of the apoptosis-related gene *CASP3* was significantly elevated at d 70 (*P* < 0.05; Fig. 1H).

### Age-related changes in crop mucosal barrier and immune status

**Fig. 2:**
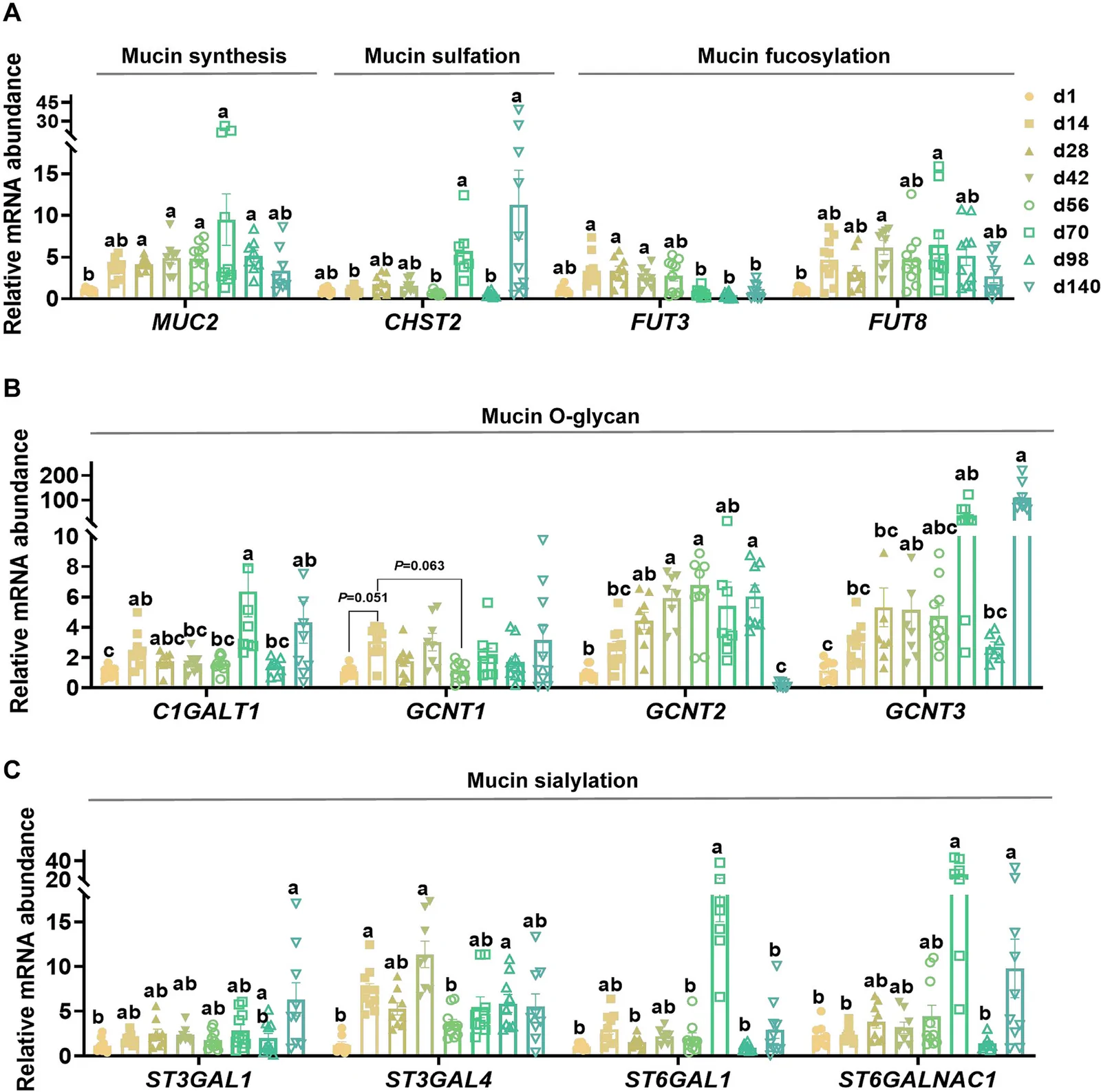
Development of mucin synthesis and modification in the chicken crop across different ages. **A** Relative mRNA expression of *MUC2* and sulfation (*CHST2*) and fucosylation (*FUT8, FUT3*) genes. **B** Relative mRNA expression of O-glycan synthesis genes (*C1GALT1, GCNT1, GCNT2, GCNT3*). **C** Relative mRNA expression of sialylation genes (*ST3GAL1, ST3GAL4, ST6GALNAC1, ST6GAL1*). Data are expressed as mean ± SEM. Statistical significance was assessed by one-way ANOVA. Different letters (**a**–**c**) indicate significant differences among the treatment groups (*P* < 0.05). *C1GALT1*, Core 1 beta-1,3-galactosyltransferase; *CHST2*, Carbohydrate sulfotransferase 2; *FUT3*, Fucosyltransferase 3; *FUT8*, Fucosyltransferase 8; *GCNT1*, Glucosaminyl (N-acetyl) transferase 1; *GCNT2*, Glucosaminyl (N-acetyl) transferase 2; *GCNT2*, Glucosaminyl (N-acetyl) transferase 3; *MUC2*, Mucin 2; SEM, Standard error of the mean; *ST3GAL1*, ST3 beta-galactoside alpha-2,3-sialyltransferase 1; *ST3GAL4*, ST3 beta-galactoside alpha-2,3-sialyltransferase 4; *ST6GAL1*, ST6 beta-galactoside alpha-2,6-sialyltransferase 1; *ST6GALNAC1*, ST6 N-acetylgalactosaminide alpha-2,6-sialyltransferase 1; *WNT3*, Wnt family member 3

Developmental changes in the crop mucosal barrier were analyzed at the levels of mucin synthesis and mucin modification. The expression of the representative mucin synthesis gene *MUC2* increased significantly from d 1 to d 28 (*P* < 0.05) and remained relatively stable thereafter (Fig. 2A). Genes involved in sulfation, including *CHST2*, showed significant variation across developmental stages but did not exhibit a clear temporal expression pattern (*P* < 0.05; Fig. 2A). Among the fucosylation-related genes, *FUT3* expression was significantly higher at d 14 to d 28 than at d 70 to d 140 (*P* < 0.05; Fig. 2A), whereas *FUT8* expression increased progressively from d 1 to d 42 and remained relatively stable thereafter (*P* < 0.05; Fig. 2A). Among the O-glycan modification–related genes, the expression of *C1GALT1* and *GCNT1* gradually increased from d 1 to d 14 and then remained relatively stable (*P* < 0.05; Fig. 2B), whereas the expression of *GCNT2* and *GCNT3* increased progressively from d 1 to d 42 and remained relatively stable thereafter (*P* < 0.05; Fig. 2B). Among the sialylation-related genes, *ST3GAL1* showed significant variation across developmental stages but did not exhibit a clear temporal expression pattern (*P* < 0.05; Fig. 2C). *ST6GAL1* and *ST6GALNAC1* showed significantly higher expression only at d 70 (*P* < 0.05; Fig. 2C), whereas *ST3GAL4* expression increased progressively from d 1 to d 42, decreased significantly between d 42 and d 56, and subsequently showed a slight recovery (*P* < 0.05; Fig. 2C).

sIgA was analyzed as an indicator of mucosal immune status in the crop. sIgA levels increased from d 1 to d 28, then fluctuated between d 28 and d 70, and reached a relatively high and stable level from d 98 to d 140, with the highest level observed at d 140 (*P* < 0.05; Fig. 1G).

### Age-related changes in crop microbiota structure

**Fig. 3:**
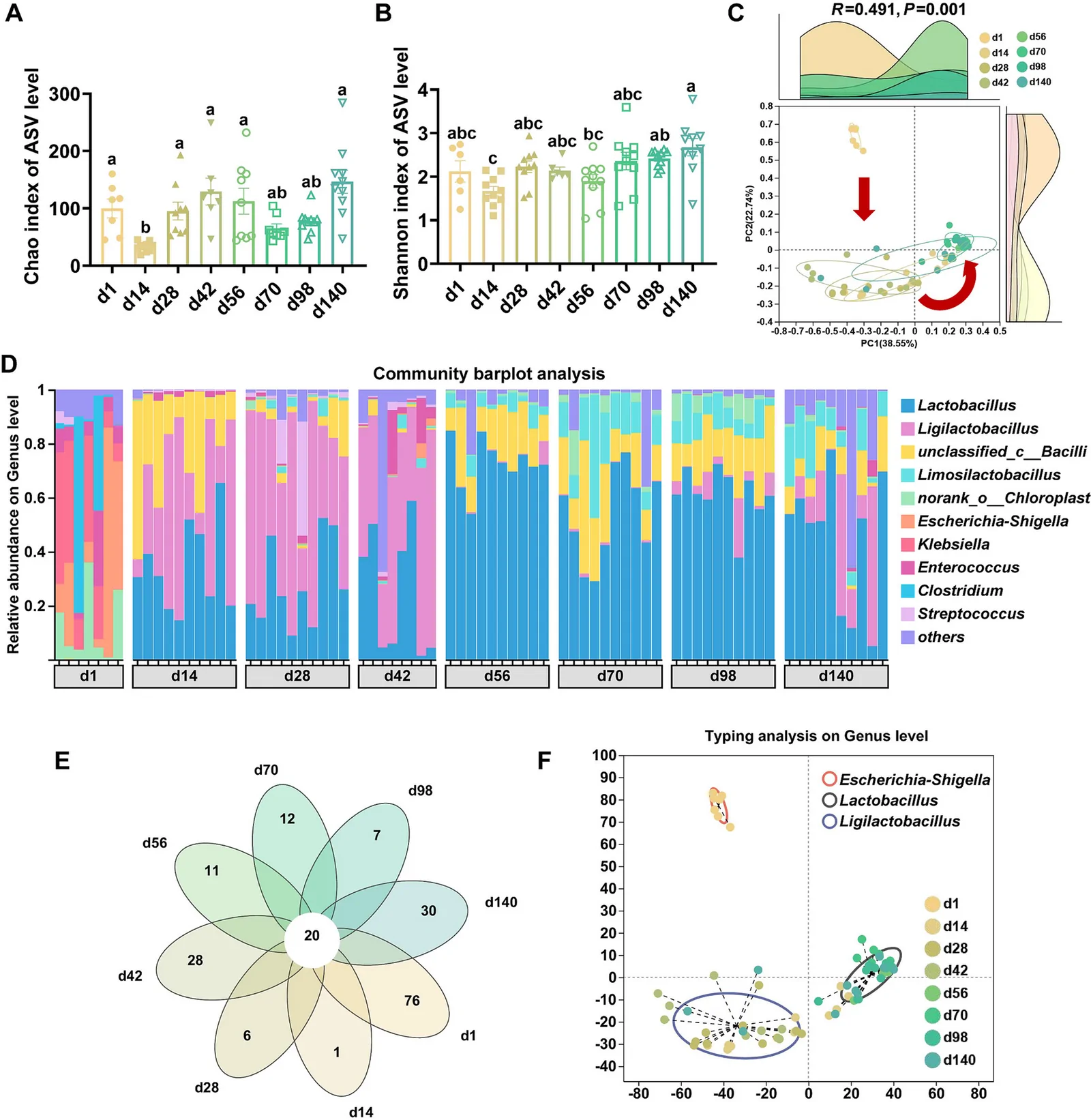
Development of microbial community structure in the chicken crop across different ages. **A** and **B** Chao index and Shannon index of the crop microbiota. **C** Beta diversity of the crop microbiota based on Bray–Curtis dissimilarity analysis. **D** Relative abundance of the top 10 bacterial genera. **E** Venn diagram of the crop microbiota. **F** Analysis of the type of crop microbiota at the genus level. Data are expressed as mean ± SEM. Statistical significance was assessed by one-way Different letters (**a**–**c**) indicate significant differences among the treatment groups (*P* < 0.05). SEM, Standard error of the mean

The diversity parameters of the crop microbiota were first assessed. Alpha diversity analysis showed that the Chao1 index decreased at d 14 (Fig. 3A; *P* < 0.05), followed by a recovery and stabilization, while the Shannon index increased progressively and peaked at d 140 (Fig. 3B; *P* < 0.05). Beta diversity analysis revealed three age-associated clusters: d 1, d 14 to 42, and d 56 to 140 (Fig. 3C; *P* < 0.05).

**Fig. 4:**
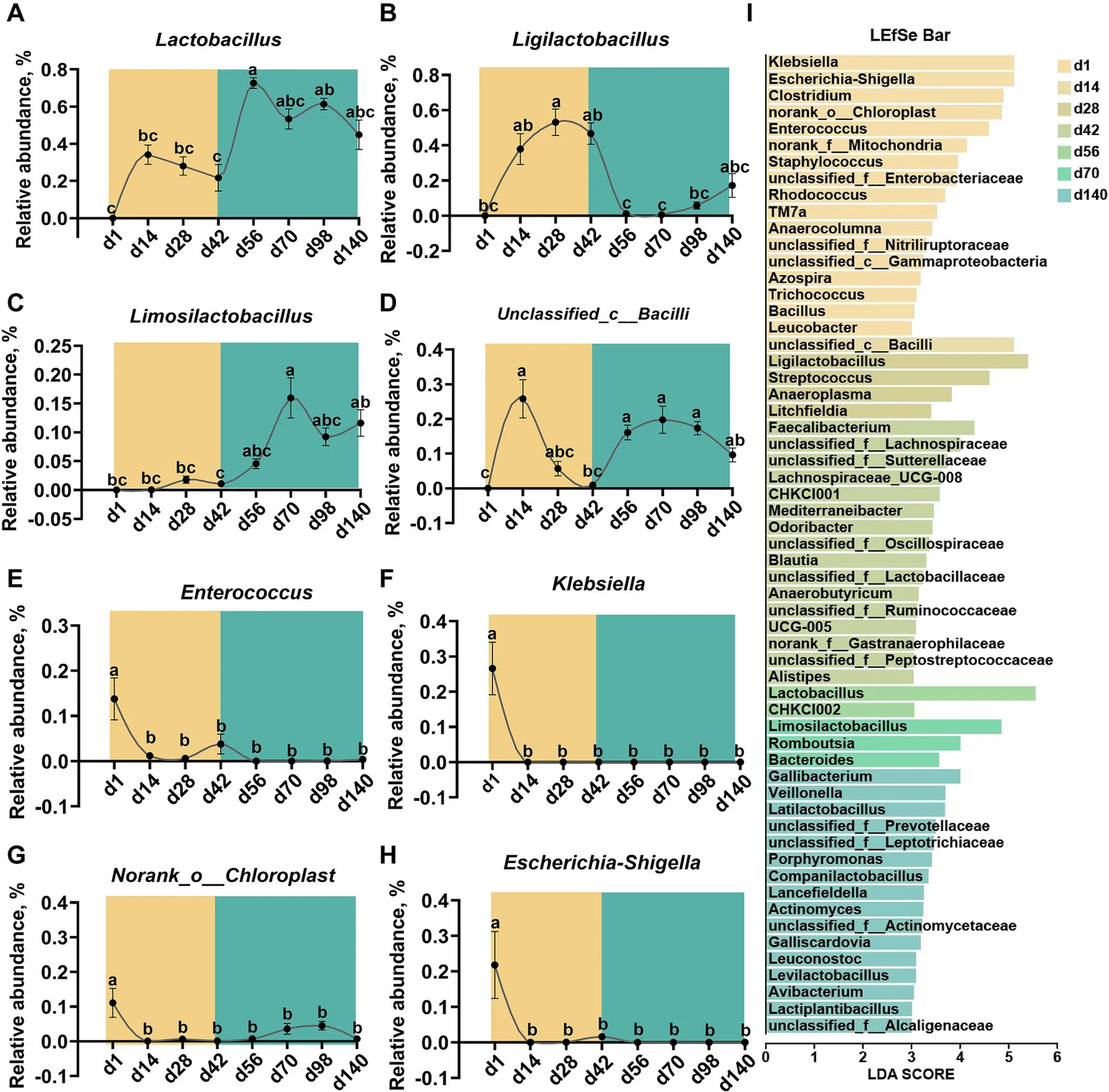
Differential microbial taxa in the chicken crop across different developmental stages. **A**–**H** Changes in the relative abundance of the eight bacterial genera. **I** Differences in bacterial genera across eight time points identified by LEfSe analysis. Data were analyzed using LEfSe analysis (LDA score > 2, *P* < 0.05). Data are expressed as mean ± SEM. Statistical significance was assessed by one-way ANOVA. Different letters (**a**–**c**) indicate significant differences among the treatment groups (*P* < 0.05). LDA, Linear discriminant analysis; LEfSe, Linear discriminant analysis effect size. SEM, Standard error of the mean

Furthermore, the composition of the crop microbiota was characterized, showing the relative abundance of the top 10 genera (Fig. 3D). A Venn diagram revealed that 20 genera were shared across the eight age groups, while age-specific genera numbered 76 at d 1, 1 at d 14, 6 at d 28, 28 at d 42, 11 at d 56, 12 at d 70, 7 at d 98, and 30 at d 140 (Fig. 3E). PAM clustering based on genus-level relative abundance profiles classified the crop microbiota into three types, each defined by its dominant genus (Top 1 genus): d 1 (*Escherichia–Shigella* type), d 14 to d 42 (*Lactobacillus* type), and d 56 to d 140 (*Ligilactobacillus* type). Specifically, at d 1, the microbiota was characterized by *norank_o__Chloroplast*, *Escherichia–Shigella*, *Klebsiella*, and *Enterococcus* (Fig. 4A–H). At d 14, the community was dominated by *Lactobacillus*, *Ligilactobacillus*, and *unclassified_c__Bacilli* (Fig. 4A–H). At d 28 and d 42, *Lactobacillus* and *Ligilactobacillus* remained predominant, accompanied by a small proportion of *unclassified_c__Bacilli* (Fig. 4A–H). From d 56 to d 140, the community was mainly characterized by *Lactobacillus*, *unclassified_c__Bacilli*, and *Limosilactobacillus* (Fig. 4A–H). LEfSe analysis further supported the stage-specific microbial signatures identified based on the genus-level relative abundance analysis (Fig. 4I).

### Age-related changes in predicted crop microbiota functions

**Fig. 5:**
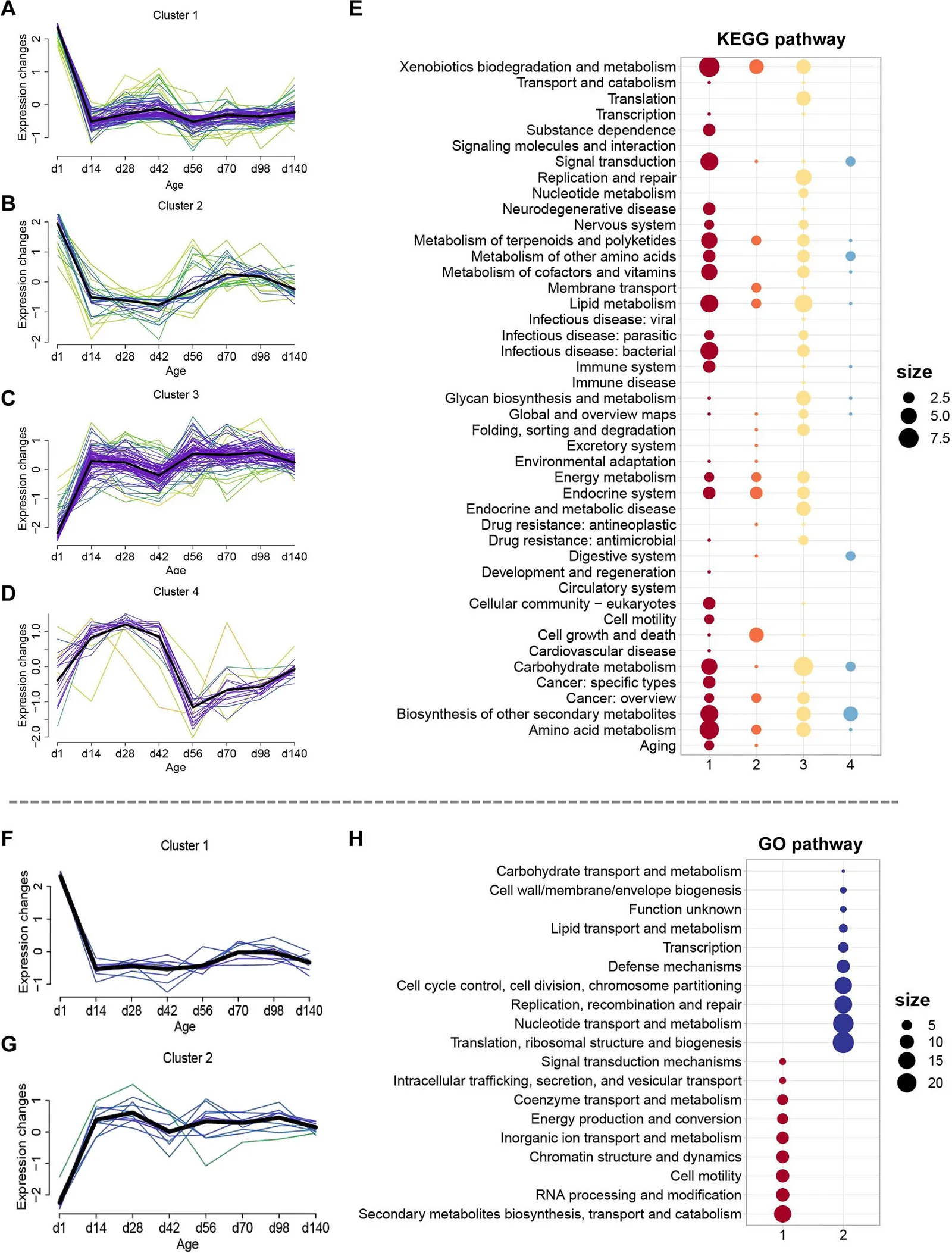
Predicted functional profiles of the crop microbiota across different ages in chickens. **A**–**D** KEGG pathway clustering of microbial predicted function (level 3). **E** Numbers of KEGG pathways in each cluster (level 3). The area of the circles represents the number of pathways. **F** and **G** GO functional clustering of microbial predicted function. **H** Numbers of GO functions in each cluster. The area of the circles represents −log_10_ (adjusted *P* value). KEGG and GO enrichment analyses were performed using PICRUSt2. Cluster analysis was assessed by Mfuzz. Differentially enriched KEGG and GO pathways were identified using one-way ANOVA implemented in the limma package, with adjusted *P* < 0.05 considered statistically significant. GO, Gene Ontology; KEGG, Kyoto Encyclopedia of Genes and Genomes; PICRUSt2, Phylogenetic Investigation of Communities by Reconstruction of Unobserved States 2

Predicted microbial functions in the developing crop were analyzed using PICRUSt2, and age-related dynamics of KEGG and GO pathways from d 1 to d 140 were characterized (Fig. 5). Mfuzz clustering identified four temporal patterns for KEGG pathways (Fig. 5A–E) and two patterns for GO terms (Fig. 5F–H).

At d 1, predicted functions were enriched in pathways related to microbial motility and colonization, including bacterial chemotaxis, flagellar assembly, and biofilm formation (Fig. 5E and H). In parallel, several pathways linked to host interaction and immune-related processes were enriched, such as IL-17 signaling and bacterial infectious disease pathways. Early-stage metabolic signatures were also evident, including representative functions in amino acid metabolism (e.g., phenylalanine and lysine metabolism), carbohydrate metabolism (e.g., glyoxylate and dicarboxylate metabolism and the citrate cycle), and vitamin/cofactor metabolism (e.g., folate and vitamin B_6_ metabolism), together with secondary metabolite and xenobiotic metabolism. Consistently, GO analysis showed higher enrichment at d 1 than at d 14 to d 140 in cell motility, intracellular trafficking/secretion, and coenzyme and inorganic ion transport and metabolism.

From d 14 onward, predicted functions shifted toward core metabolic activity and growth-related processes, as indicated by higher enrichment in translation, replication and repair, and folding/sorting/degradation, together with broad nutrient metabolism (Fig. 5E and H). Notably, during d 14 to d 42, several pathways associated with carbohydrate and fatty acid metabolism showed prominent enrichment patterns, including fructose and mannose metabolism, butanoate metabolism, and fatty acid metabolism, accompanied by glycan metabolism and biotin metabolism.

In later stages (d 56 to d 140), predicted functions were characterized by increased enrichment in pathways related to transport systems and metabolic maturation (Fig. 5E and H). This included membrane transport functions such as ABC transporters and the bacterial secretion system, alongside multiple nutrient metabolic pathways involving amino acid, carbohydrate, and lipid metabolism. GO terms supported these trends, with higher enrichment in cell wall/membrane/envelope biogenesis, carbohydrate transport and metabolism, and lipid transport and metabolism.

## Discussion

The crop regulates feed intake in poultry, and its microbial fermentation products have the potential to promote intestinal health, thereby influencing production performance [3, 5]. However, current research has mainly focused on specific physiological stages, and a comprehensive understanding of crop function remains limited [36–38]. Therefore, this study used slow-growing chickens with clearly defined developmental stages to characterize the developmental trajectories of crop histomorphology, microbiota, and mucosal immune status. Crop development was divided into two stages: the establishment stage (d 1 to d 42) and the remodeling stage (d 42 to d 140). The characteristics and potential implications of these stages are discussed below.

The period from d 1 to d 42 represented a critical window for the establishment of crop structure and the microbial community. At hatch, chicks had not yet initiated feed intake, and the crop lumen had only recently been exposed to the external environment. At hatching, chicks exhibited relatively high serum TC and TG levels, likely reflecting their reliance on yolk sac-derived lipids before feed intake [39, 40]. At this stage, the expression of genes associated with tissue development (*WNT3*), mucin synthesis and glycosylation, together with the level of sIgA, remained low. The crop microbiota was dominated by environmentally derived facultative anaerobes, including *Escherichia–Shigella*, *Klebsiella*, and *Enterococcus*. Correspondingly, the predicted microbial functions were mainly enriched in pathways related to bacterial motility, chemotaxis, flagellar assembly, and biofilm formation, indicating that the crop microbiota was undergoing active colonization. This pattern is consistent with the early establishment of the intestinal microbiota in poultry [41]. Therefore, early access to feed after hatch [42, 43], together with improved environmental hygiene [44], may facilitate crop development and the healthy establishment of the crop microbiota. From d 1 to d 14, following the initiation of feed intake, serum TC and TG levels declined, whereas serum glucose level increased transiently before declining and eventually stabilizing, reflecting metabolic adaptation to rapid growth and development [39, 40]. Meanwhile, the continuous entry of feed into the crop provided persistent mechanical stimulation, which may have promoted crop tissue growth, as evidenced by the progressive increases in crop weight and muscular layer thickness, accompanied by a significant upregulation of *WNT3* expression [43]. As host development and microbial colonization progressed simultaneously [45, 46], the crop mucosal layer became thicker, and the expression of genes involved in mucin glycosylation was markedly increased, including genes associated with fucosylation (*FUT3* and *FUT8*), O-glycan modification (*C1GALT1*, *GCNT1*, *GCNT2*, and *GCNT3*), and sialylation (*ST3GAL4*). In addition, the level of sIgA in the crop tissue increased progressively with the initiation of feed intake and microbial colonization [47, 48]. The maturation of the mucosal barrier together with enhanced local mucosal immunity may have contributed to ecological selection within the crop, promoting a gradual transition of the microbial community from environmentally derived facultative anaerobes to a community dominated by lactic acid bacteria [15]. In addition, fermentation of dietary carbohydrates by lactic acid bacteria progressively lowered the crop pH, thereby creating a microenvironment favorable for acid-tolerant lactobacilli while limiting the persistence of some environmentally derived bacteria [49]. This positive feedback between host-mediated ecological selection and microbial metabolism likely facilitated the establishment of a lactobacilli-dominated community. Consequently, by d 14, the crop microbiota was primarily composed of *Lactobacillus*, *Ligilactobacillus*, and *unclassified Bacilli*. *Lactobacillus* and *Ligilactobacillus* are well-recognized probiotic genera with strong adhesion capacity, efficient carbohydrate fermentation, acid production, and antimicrobial activity [11, 12, 50]. Owing to the limited taxonomic resolution of 16S rRNA gene sequencing, the *unclassified Bacilli* could not be further assigned to lower taxonomic levels. This group may include members of Lactobacillaceae, Enterococcaceae, Streptococcaceae, and Bacillaceae, and its precise taxonomic composition and ecological functions require further investigation using metagenomic sequencing or bacterial isolation and cultivation. Consistent with these compositional changes, the predicted microbial functions shifted from pathways associated with early colonization to those involved in carbohydrate metabolism, fatty acid metabolism, and butyrate metabolism, suggesting the progressive maturation of the fermentative capacity of the crop microbiota. From d 14 to d 42, crop weight continued to increase, whereas the thickness of both the mucosal and muscle layers gradually reached a stable level. The expression of genes involved in mucin glycosylation and the concentration of sIgA increased further, indicating continued maturation of the crop mucosal barrier and local mucosal immune status. During the same period, *Lactobacillus* and *Ligilactobacillus* became increasingly dominant, whereas the abundance of *unclassified Bacilli *progressively declined. Notably, *unclassified Bacilli* increased rapidly between d 1 and d 14 but nearly disappeared between d 14 and d 42. This observation suggests that these bacteria may primarily participate in early niche occupation during microbial community establishment. As the crop environment matured, characterized by lower luminal pH, a more developed mucosal barrier, and the continuous expansion of dominant lactobacilli, their ecological niche may have gradually been replaced by *Lactobacillus* and *Ligilactobacillus* [41, 51]. The developmental trajectory of the crop microbiota observed in the present study was generally consistent with previous reports and further expands our understanding of microbial succession in the chicken crop [18, 52, 53]. Interestingly, this period also coincides with the critical window for intestinal microbiota establishment in poultry [41], suggesting that the developmental trajectories of the crop and intestinal microbiota may be synchronized to some extent [19]. Collectively, the period from d 1 to d 42 represents a critical developmental window characterized by rapid structural development of the crop, progressive maturation of the mucosal barrier and local mucosal immunity, and coordinated establishment of the crop microbiota. Targeted interventions during this stage may therefore contribute to gastrointestinal development and improved production performance.

The period from d 42 to d 140 represented the functional remodeling stage of the crop. During this period, crop weight increased rapidly before gradually reaching a stable level after d 70, accompanied by decreased expression of the developmental gene *WNT3* and increased expression of the apoptosis-related gene *CASP3*. The rapid increase in crop weight coincided with the period of rapid body weight gain, suggesting that enlargement of the crop may facilitate the increased nutritional demands associated with host growth [54]. However, the mechanisms by which host development regulates rapid crop enlargement remain unclear and warrant further investigation. As host development continued, birds were transitioned to a grower diet after d 42, and the crop mucosa underwent further remodeling. Specifically, the expression of the sialylation-1 related gene *ST3GAL4* decreased markedly between d 42 and d 56 before gradually recovering. In addition, *FUT3* expression remained significantly lower than that observed from d 14 to d 28, whereas the expression levels of *FUT8*, *GCNT2*, *GCNT3*, and *ST3GAL4* were generally comparable to those during d 14 to d 28. Crop tissue sIgA levels also increased further during this stage, which may reflect the combined effects of continued maturation of local mucosal immunity and persistent microbial colonization [15, 55]. Collectively, these findings indicate that the crop mucosa continued to mature throughout this developmental period. The relationships between specific mucin glycosylation modifications and individual microbial taxa remain poorly understood, and the underlying regulatory mechanisms require further investigation. The crop microbial community also underwent further succession during this period, which was likely influenced by both changes in host physiological status and the dietary transition [56]. Consequently, the relative abundance of *Ligilactobacillus* declined rapidly between d 42 and d 56 and became nearly undetectable thereafter, whereas *Lactobacillus*, *unclassified Bacilli*, and *Limosilactobacillus* gradually increased and remained relatively stable from d 56 to d 140. These compositional changes occurred concurrently with alterations in mucin glycosylation patterns, suggesting that continued maturation of the crop mucosal environment may provide favorable conditions for further microbial community remodeling [46]. Consistent with these compositional changes, the predicted microbial functions were enriched in membrane transport systems and multiple nutrient metabolism pathways, suggesting the progressive establishment of a more mature fermentative metabolic capacity within the crop microbiota. These functional changes may enhance microbial nutrient metabolism and utilization, thereby supporting host growth. Notably, *unclassified Bacilli* reappeared during this stage, suggesting that these taxa may participate in nutrient metabolism and fermentation processes [56–58], although their precise ecological roles remain to be elucidated. Overall, the period from d 42 to d 140 represents an important developmental stage characterized by continued structural and functional remodeling of the crop together with further reconstruction of the microbial community.

## Conclusion

This study systematically characterized the stage-specific development of the crop in poultry. The results demonstrated that d 1 to d 42 represents a critical period for structural development and microbial colonization, whereas d 42 to d 140 is characterized by rapid crop expansion and microbial remodeling, accompanied by a gradual increase and eventual stabilization of sIgA levels. Crop development appears to be a coordinated process regulated by host structural maturation, microbial succession, and nutritional factors. These findings define key temporal windows for crop functional development and provide a theoretical basis for targeted regulation aimed at improving crop function and ultimately enhancing poultry production performance.

## Supplementary Information

Additional file 1: Table S1. Alpha diversity of crop microbiota. Table S2. Top 50 genus of crop microbiota. Table S3. KEGG prediction function of crop microbiota. Table S4. GO prediction function of crop microbiota. Table S5. Microbiota 16S rRNA analysis.

## Data Availability

The data produced or analyzed during the current study are available from the corresponding author by reasonable request.

## Abbreviations

AB-PAS: Alcian blue–periodic acid Schiff
ALB: Albumin
ALT: Alanine aminotransferase
AST: Aspartate aminotransferase
ASV: Amplicon sequence variant
CASP3: Caspase 3
C1GALT1: Core 1 beta-1,3-galactosyltransferase
CHST2: Carbohydrate sulfotransferase 2
FUT3: Fucosyltransferase 3
FUT8: Fucosyltransferase 8
GCNT1: Glucosaminyl (N-acetyl) transferase 1
GCNT2: Glucosaminyl (N-acetyl) transferase 2
GCNT3: Glucosaminyl (N-acetyl) transferase 3
GLU: Glucose
GO: Gene Ontology
H&E: Hematoxylin and eosin
KEGG: Kyoto Encyclopedia of Genes and Genomes
LDA: Linear discriminant analysis
LEfSe: Linear discriminant analysis effect size
MUC2: Mucin 2
One-way ANOVA: One-way analysis of variance
PAM: Partitioning around medoids
PCoA: Principal coordinate analysis
PICRUSt2: Phylogenetic Investigation of Communities by Reconstruction of Unobserved States 2
SEM: Standard error of the mean
SIgA: Secretory immunoglobulin A
ST3GAL1: ST3 beta-galactoside alpha-2,3-sialyltransferase 1
ST3GAL4: ST3 beta-galactoside alpha-2,3-sialyltransferase 4
ST6GAL1: ST6 beta-galactoside alpha-2,6-sialyltransferase 1
ST6GALNAC1: ST6 N-acetylgalactosaminide alpha-2,6-sialyltransferase 1
TBIL: Total bilirubin
TC: Total cholesterol
TG: Triglyceride
TP: Total protein
UA: Uric acid
WNT3: Wnt family member 3
