# Supplementary material for: Developmental dynamics of crop morphology, microbiota, and mucosal immunity in chickens
Source: J Anim Sci Biotechnol. 2026 Sep 8;17:174. doi: 10.1186/s40104-026-01495-8 (PMC13551962; doi:10.1186/s40104-026-01495-8)
Supplement: Supplementary file 1 [file 40104_2026_1495_MOESM1_ESM.zip › Table S5. Microbiota 16S rRNA analysis (PCR amplification).docx]

**Intestinal microbiota 16S rRNA analysis**

**(PCR amplification results)**

1. Gel image of PCR amplification results

| **Number** | **Intestinal area** | **Treat** | **Number** | **Intestinal area** | **Treat** |
| --- | --- | --- | --- | --- | --- |
| 1 | Crop | day1-1 | 51 | Crop | day70-2 |
| 2 | Crop | day1-2 | 52 | Crop | day70-3 |
| 3 | Crop | day1-3 | 53 | Crop | day70-4 |
| 4 | Crop | day1-4 | 54 | Crop | day70-5 |
| 5 | Crop | day1-5 | 55 | Crop | day70-6 |
| 6 | Crop | day1-6 | 56 | Crop | day70-7 |
| 7 | Crop | day1-7 | 57 | Crop | day70-8 |
| 8 | Crop | day1-9 | 58 | Crop | day70-9 |
| 9 | Crop | day1-10 | 59 | Crop | day70-10 |
| 10 | Crop | day14-1 | 60 | Crop | day84-1 |
| 11 | Crop | day14-2 | 61 | Crop | day84-2 |
| 12 | Crop | day14-3 | 62 | Crop | day84-3 |
| 13 | Crop | day14-4 | 63 | Crop | day84-4 |
| 14 | Crop | day14-5 | 64 | Crop | day84-5 |
| 15 | Crop | day14-6 | 65 | Crop | day84-6 |
| 16 | Crop | day14-7 | 66 | Crop | day84-7 |
| 17 | Crop | day14-8 | 67 | Crop | day84-8 |
| 18 | Crop | day14-9 | 68 | Crop | day84-9 |
| 19 | Crop | day14-10 | 69 | Crop | day84-10 |
| 20 | Crop | day28-1 | 70 | Crop | day98-1 |
| 21 | Crop | day28-2 | 71 | Crop | day98-2 |
| 22 | Crop | day28-3 | 72 | Crop | day98-3 |
| 23 | Crop | day28-4 | 73 | Crop | day98-4 |
| 24 | Crop | day28-5 | 74 | Crop | day98-5 |
| 25 | Crop | day28-6 | 75 | Crop | day98-6 |
| 26 | Crop | day28-7 | 76 | Crop | day98-7 |
| 27 | Crop | day28-8 | 77 | Crop | day98-8 |
| 28 | Crop | day28-9 | 78 | Crop | day98-9 |
| 29 | Crop | day28-10 | 79 | Crop | day98-10 |
| 30 | Crop | day42-1 | 80 | Crop | day112-1 |
| 31 | Crop | day42-2 | 81 | Crop | day112-2 |
| 32 | Crop | day42-3 | 82 | Crop | day112-3 |
| 33 | Crop | day42-4 | 83 | Crop | day112-4 |
| 34 | Crop | day42-5 | 84 | Crop | day112-5 |
| 35 | Crop | day42-6 | 85 | Crop | day112-6 |
| 36 | Crop | day42-7 | 86 | Crop | day112-7 |
| 37 | Crop | day42-8 | 87 | Crop | day112-8 |
| 38 | Crop | day42-9 | 88 | Crop | day112-9 |
| 39 | Crop | day42-10 | 89 | Crop | day112-10 |
| 40 | Crop | day56-1 | 90 | Crop | day140-1 |
| 41 | Crop | day56-2 | 91 | Crop | day140-2 |
| 42 | Crop | day56-3 | 92 | Crop | day140-3 |
| 43 | Crop | day56-4 | 93 | Crop | day140-4 |
| 44 | Crop | day56-5 | 94 | Crop | day140-5 |
| 45 | Crop | day56-6 | 95 | Crop | day140-6 |
| 46 | Crop | day56-7 | 96 | Crop | day140-7 |
| 47 | Crop | day56-8 | 97 | Crop | day140-8 |
| 48 | Crop | day56-9 | 98 | Crop | day140-9 |
| 49 | Crop | day56-10 | 99 | Crop | day140-10 |
| 50 | Crop | day70-1 |  |  |  |

**Note**: In this study, certain tissue samples were lost and, therefore, were not included in further analysis. The specific sample numbers are as follows: Day 1 (samples 2, 8, 9), Day 42 (samples 3, 10), Day 84 (all samples), and Day 112 (all samples).
